# Graphlet characteristics in directed networks

**DOI:** 10.1038/srep37057

**Published:** 2016-11-10

**Authors:** Igor Trpevski, Tamara Dimitrova, Tommy Boshkovski, Nikola Stikov, Ljupcho Kocarev

**Affiliations:** 1 Macedonian Academy of Sciences and Arts, Skopje, Republic of Macedonia; 2 Institute for Biomedical Engineering, Ecole Polytechnique, Montreal, QC, Canada; 3 Montreal Heart Institute, Montreal, QC, Canada; 4 Faculty of Computer Science and Engineering, UKIM, Skopje, Republic of Macedonia; 5 BioCircuits Institute, UC San Diego, La Jolla, CA 92093-0402, USA

## Abstract

Graphlet analysis is part of network theory that does not depend on the choice of the network null model and can provide comprehensive description of the local network structure. Here, we propose a novel method for graphlet-based analysis of directed networks by computing first the *signature vector* for every vertex in the network and then the *graphlet correlation matrix* of the network. This analysis has been applied to brain effective connectivity networks by considering both direction and sign (inhibitory or excitatory) of the underlying directed (effective) connectivity. In particular, the signature vectors for brain regions and the graphlet correlation matrices of the brain effective network are computed for 40 healthy subjects and common dependencies are revealed. We found that the signature vectors (node, wedge, and triangle degrees) are dominant for the excitatory effective brain networks. Moreover, by considering only those correlations (or anti correlations) in the correlation matrix that are significant (>0.7 or <−0.7) and are presented in more than 60% of the subjects, we found that excitatory effective brain networks show stronger causal (measured with Granger causality) patterns (G-causes and G-effects) than inhibitory effective brain networks.

The complexity of systems is frequently the result of non-trivial local connectivity and interaction of its constituents parts. A number of network structural characteristics have recently been the subject of particularly intense research, including degree distributions^1^, community structure^2^,^3^, and various measures of vertex centrality^4^,^5^, to mention only a few. Vertices may have attributes associated with them; for example, properties of proteins in protein-protein interaction networks^6^, users’ social network profiles^7^, or authors’ publication histories in co-authorship networks^8^. Two approaches that focus on the local connectivity of subgraphs within a network are Motifs and Graphlets. Motifs are defined as sub-graphs that repeat frequently in the networks i.e they repeat at frequency higher than in the random graphs^9^,^10^, and they depend on the choice of the network’s null model. In contrast, graphlets are induced sub-graphs of a network that appear at any frequency and hence are independent of a null model. They have been introduced recently^11^ and they have found numerous applications as building blocks of network analysis in various disciplines ranging from social science^12^,^13^ to biology^14^,^15^. In social science, graphlet analysis (known as sub-graph census) is widely adopted in sociometric studies^12^. Much of the work in this vein focused on analyzing triadic tendencies as important structural features of social networks (e.g., transitivity or triadic closure) as well as analyzing triadic configurations as the basis for various social network theories (e.g., social balance, strength of weak ties, stability of ties, or trust^16^). In biology graphlets were used to infer protein structure^17^, to compare biological networks^14^,^15^, and to characterize the relationship between disease and structure of networks^18^.

Many of the real-world networks are directed, but until now no method has been proposed based on graphlets that can provide information about local structure of directed networks. Here, we offer a graphlet-based approach for analysis of the local structure of a directed network. In the method proposed in this manuscript, we compute for each vertex, a vector of structural features, called *signature vector*, based on the number of graphlets associated with the vertex, and for the network its *graphlet correlation matrix*, measuring graphlet dependencies which reveal unknown organizational principles of the network. We applied the technique to brain effective networks of 40 healthy subjects, and we found that many of the subjects share similar patterns in their network’s local structure. In brain networks a node is associated with different types of elements, depending on the level of interest in the brain, and an edge represents the connection or interaction between two elements^19^. If the brain is studied on a macro level then there are three types of connectivity: i) structural (referring to anatomical links); ii) functional (capturing the patterns of deviations from statistical independence between time series from distributed brain regions) and iii) effective connectivity, which can be considered as the union of the previous two, and it describes the causal effects between neural elements. Structural and functional brain connectivity usually are modeled as undirected networks despite of effective connectivity which is modeled as directed network. Effective connectivity is defined as the influence one node (neuronal population) exerts over another, under a particular network model of causal dynamics. Neither structural nor functional connectivity in large-scale networks specify the direction or sign (inhibitory or excitatory) of underlying directed (effective) connectivity. Directionality in effective brain networks originates from their definition. The causal effect between two neural elements A and B is not symmetric i.e. the causal effect of A over B can be greater or smaller than the causal effect of B over A. Effective connectivity is becoming increasingly important in the analysis of functional integration because the underlying model defines the mechanisms of neuronal coupling^20^. We found that regions (nodes) in excitatory effective brain networks not only have larger signature vectors than inhibitory effective brain networks but also have richer local structure in terms of Granger causalities (G-causes and G-effects) of (1-hop and 2-hop) neighboring regions.

## Methods

Some of the more traditional approaches for studying the global structure are the degree distribution, network diameter and clustering coefficients, while on the other hand a new bottom-up approach has been introduced that focuses on small subgraphs (subnetworks whose nodes and edges belong to the large network) that appear frequently, called network motifs. Similar definition is found for graphlets, which were defined for undirected networks in Przulj^14^,^15^ and represent small induced sub-graphs of a large network that appear at any frequency and hence, are independent of a null model. Here we give a definition of graphlets for directed networks and using 2-node and 3-node graphlets we propose a definition of a signature vector for each vertex.

### Graphlet definition

Let *G* = (*V*, *E*) be a simple directed (un-weighted) graph and let **A** = [*a*_*ij*_] be its *n* × *n* adjacency matrix, where *n* is the number of vertices, such that *a*_*ii*_ = 0 (self-arcs are excluded). For an element of the edge set *E* we write (*i*, *j*) to denote *i* → *j*. Directed graphs are treated as having two different types of edges: directed and reciprocal. The research on reciprocal edges originates with the triad census work of Holland and Leinhardt^12^. A reciprocal edge is technically a pair of directed edges, {(*i*, *j*), (*j*, *i*)}, that we treat as a single reciprocal edge.

A graphlet *G*_*k*_ = (*V*_*k*_, *E*_*k*_) is an induced sub-graph that consists of a subset of *k* vertices of the graph *G* = (*V*, *E*) (i.e., *V*_*k*_ ⊂ *V*) together with all the edges whose endpoints are both in this subset (i.e., *E*_*k*_ = {*e* ∈ *E*:*e* = (*u*, *v*) *and u*, *v* ∈ *V*_*k*_}). Here, we consider only weakly connected graphlets.

For a node *i* of network *G*, the automorphism orbit of *i* is the set of nodes of *G* that can be mapped to *i* by an automorphism, an isomorphism of a network with itself; i.e., a bijection of nodes that preserves node adjacency. Automorphism orbits have been used to generalize the graph degree distribution^14^,^15^. Given a family of graphlets of size *k* nodes, 2 ≤ *k* ≤ 4, one can count the number of times a node *is touched* by an orbit. In directed graphs there are 1695 orbits for up to 4-node graphlets, see SI. The larger number of such orbits makes a complete enumeration unattractive, a problem which continues to worsen for larger graphlets. Therefore, here we restrict ourselves to working with up to 3-node graphlets, reducing the number of orbits to 48. In addition, we further reduce it to 16 orbits by considering only isomorphism triangle and wedge classes. *starting* at vertex *i* (see SI).

#### 2-node and 3-node graphlets

We define the set of in-neighbors 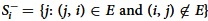, the set of out-neighbors 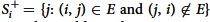, and the set of reciprocal-neighbors 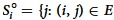 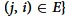. The 2-node graphlets orbits originating at a node *i* are equivalent to the out-links, in-links and reciprocal links of node *i*. The number of 2-node (out-, in-, and reciprocal-) graphlets *starting* at *i* is given by 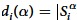, where *α* = {+, −, 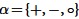}.

**Figure 1:**
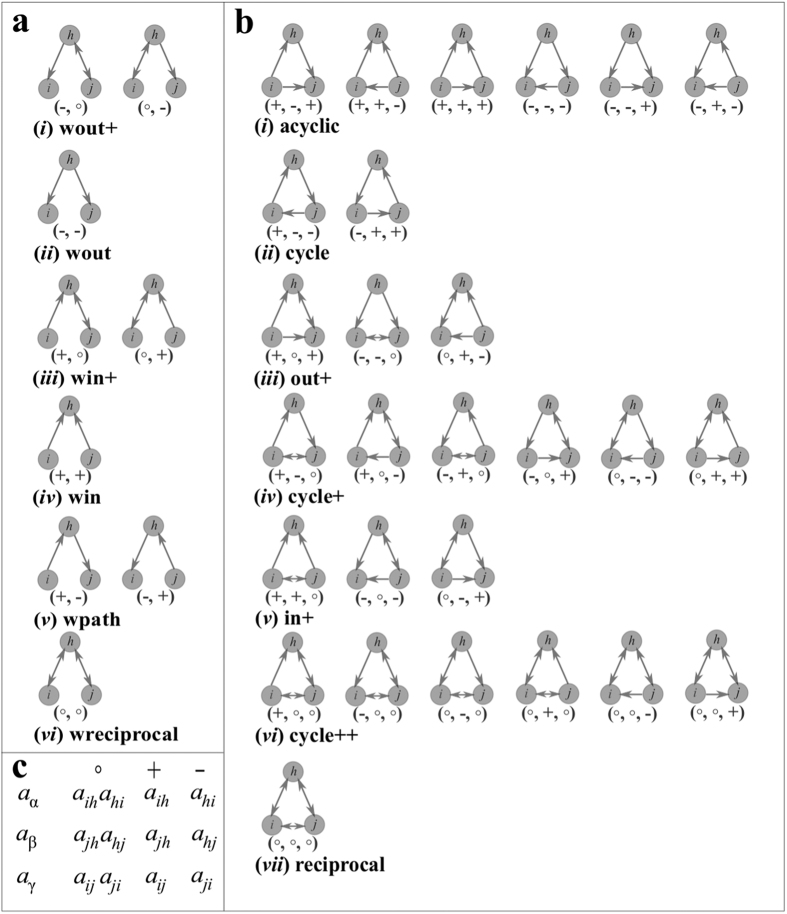
Isomorphic classes of wedges (**a**) and triangles (**b**) in directed graphs. In each wedge/triangle one vertex is labeled *i* (wedge/triangle starts at *i*). Assuming that directed (and reciprocal) edges are considered with respect to particular vertex in the wedge or the triangle (see the main text), each wedge and triangle can be labeled as (*α*, *β*) and (*α*, *β*, *γ*), respectively, where *α*, *β*, *γ* ∈ {+, −,∘}. Hence, there are 9 wedges and 27 triangles starting at *i*, which are clustered in 6 wedge isomorphic classes (**a**) and 7 triangle isomorphic classes (**b**). (**c**) Entries of adjacency matrix for out-, in-, and reciprocal-edges.

Triangle is a set of three nodes, each of which is connected to the other two. Let *T* = (*V*_*t*_, *E*_*t*_) be a triangle with nodes *i*, *h*, *j* and edges *e*_1_, *e*_2_, *e*_3_ between nodes *i* and *h*, *h* and *j*, and *i* and *j* respectively. We equip the edges with an arbitrary orientation, as this is necessary for further analysis. To be specific, we assume that directed or reciprocal character of the edges between *i* and *h* and *i* and *j* are considered with respect to the node *i* and of the edge between *h* and *j* with respect of the node *j*. Thus, for example, we write (+, −, 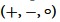) triangle to denote the triangle *T* = (*V*_*t*_, *E*_*t*_) with *V*_*t*_ = {*i*, *h*, *j*} and *E*_*t*_ = {*i* → *h*, *h* → *j*, *i* ↔ *j*}. More generally we write (*α*, *β*, *γ*) triangle to denote one of the triangle types, where *α*, *β*, *γ* = {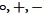, +, −}. The number of (*α*, *β*, *γ*) triangles *starting* at *i* is given by:


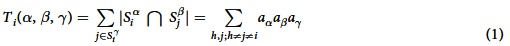


where the entries *a*_*α*_, *a*_*β*_, *a*_*γ*_ of the graph adjacency matrix are provided in Fig. 1c. Indeed, 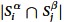 is the number of the common *α*-neighbors of *i* and *β*-neighbors of *j*. Summarizing 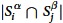 for all *j* that are *γ*-neighbors of *i*, one computes the number of (*α*, *β*, *γ*) triangles.

In a similar fashion we denote (*α*, *β*) wedges for the wedges in directed graphs. Wedge is a set of three nodes and two edges. Since directed graphs have two types of edges: directed and reciprocal, there exist 9 wedge types, which are arranged in 6 isomorphism wedge classes (Fig. 1a), and 27 triangle types, which can be grouped in 7 isomorphism triangle classes (Fig. 1b). The number of (*α*, *β*) wedges starting at vertex *i* is 
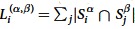
; the number of (*α*, *β*) wedges that are graphlets is given by:


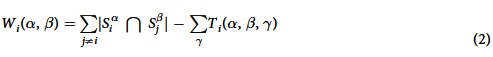


#### Signature vector

Therefore 39 quantities 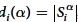, *W*_*i*_(*α*, *β*), and *T*_*i*_(*α*, *β*, *γ*) are associated with a vertex *i*. This number is further reduced to 16 by considering only isomorphic wedge and triangle classes (SI). We define a *signature/feature* vector of a vertex *i* as *F*_*i*_ = [*d*_*i*_, *W*_*i*_, *T*_*i*_]^*T*^ where:


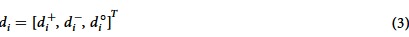



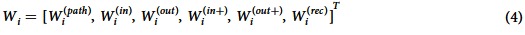



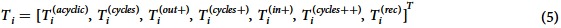



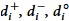
 are the numbers of 2-node graphlets, or equivalently, the out-degree, in-degree and reciprocal-edge degree of the vertex, while *W*_*i*_ and *T*_*i*_, called *wedge-degree* and *triangle-degree*, respectively, are the number of 3-node graphlets *starting at i*. Signature vectors describe only local connectivity of directed edges. Indeed, by flipping the direction of edges between all connected pairs of nodes with probability one-third (for out-, in-, and reciprocal-edges), which keeps the undirected connectivity and thus the edge density unchanged, the information about the direction of edges will be destroyed and normalized signature vectors will converge to 
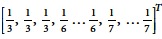
 (see SI).

### Brain networks data

In this section we provide detailed explanation of the procedure for processing the data. As previously mentioned, the data was obtained from Beijing Enhanced^21^ for a total of 40 healthy subjects. For each subject, anatomical image, resting state functional and diffusion MR images were obtained. The anatomical image presents the structural T1-weighted 3D image, the functional MR image presents the changes in the BOLD (blood-oxygen-level-dependent) signal and the diffusion MR image represents the diffusivity of water molecules in the brain.

For extraction of the effective brain networks the functional MR images were further processed. The fMRI scans were acquired during resting state of the subject, with a SIMENS Trio Tim scanner (3 Tesla, and an EPI sequence). The following parameters of the EPI sequence were used: repetition (TR)/time echo (TE) = 2000/30 ms, flip angle = 90, field of view (FOV) = 200 × 200 *mm*^2^, voxel dimension = 3 × 3 × 3 *mm*^3^, number of slices = 240.

The preprocessing of the images was performed with different techniques: at first they were corrected for slice timing and realigned for motion correction. Then the images were normalized using the MNI EPI template with affine registration followed by nonlinear transformation. Afterwards, smoothing was applied using a Gaussian Kernel of 4 mm Full Width at Half Maximum and the signal was detrended to remove any noise that may have remained from the previous steps. Finally, the images were filtered to preserve low frequency fluctuations (0.01–0.08 Hz).

The processing of the images was performed with Granger (G-) causality. Resting-State fMRI Data Analysis Toolkit (REST) was used and multivariate coefficients ROI-wise (G-) causality analysis (GCA) was adopted to generate effective brain networks. REST-GCA supports signed path coefficients multivariate GCA and integrates a batch mode coefficients computation for ROI-wise GCA^22^,^23^. The effective connectivity for each subject was reconstructed using Granger causality coefficients between the time series of 116 regions of interest (ROIs).

For each network the number of vertices (regions) is equal to 116, according to AAL (automated anatomical labeling)^24^, while the total number of edges in the networks (after pruning) is 3466 ± 43. The GCA algorithm provides positive and negative values for the effective relation between the regions. The positive value refers to the excitatory causal effect^25^,^26^, and the negative refers to inhibitory causal effect^26^,^27^. We divide the effective network considering the type of the connection and we get the network for inhibitory connectivity and the excitatory connectivity. After obtaining these networks, the weak connections were pruned using BCT toolbox [ref needed]. For each subject the proportion of the strongest weights is preserved, for both networks, meeting the following two criteria^28^,^29^,^30^: (i) 99 percent of the vertices (regions) are connected after pruning and (ii) the minimum degree of each vertex is at least 2 × ln(*N*), where *N* is the number of nodes in the network according to Network based statatistic toolbox^31^. Having two networks per subject, and 40 subjects in total, the graphlet analysis was performed.

## Results

Using this method, we measure structural similarity between vertices on directed network by considering *directed graphlets* (induced sub-graphs) associated with vertices, and, subsequently probe the structure of graphs by computing correlations of the frequencies of different graphlets present in the graph. Overall the analysis can be divided between: (1) similarity problem by computing the structural features of a vector for each vertex based on the number of graphlets associated with the vertex, and (2) for the network its graphlet correlation matrix, measuring graphlets dependencies and hence revealing unknown organizational principles of the network.

### Similarity of vertices by signature vector

For each vertex in a network, we construct its signature vector consisting of 16 coordinates corresponding to the 16 graphlets starting at the vertex. Then we construct an *N* × 16 matrix whose rows are the signature vectors for each vertex. For a given network *G*, we compute Pearson correlation coefficients between all pairs of columns of the above described matrix and present them in a 16 × 16 symmetric matrix that is termed^15^ *graphlet correlation matrix* of network *G*. In this way, the network topology and its local direction patterns, regardless of network size (the number of vertices) and network volume (the number of edges), are summarized into a 16 × 16 matrix.

**Figure 2:**
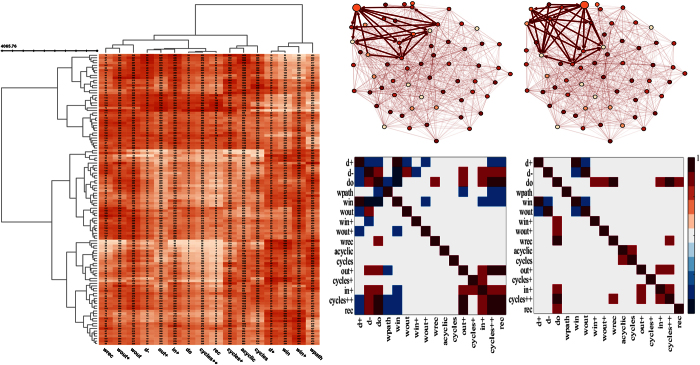
Graphlets in effective brain network. The dataset represents effective connectivity of the brain network, describing a network of directional effects of one neural region over another. (**a**) 116 regions (vertices) are considered in total (shown on the vertical axis); a directed edge represents causality of one region (vertex) over another. Each region is represented by a 16-dimensional feature vector (shown on the horizontal axis). Ward agglomerative hierarchical clustering procedure results in a heat-map representing clustered regions. Three (or four) clusters with similar local structure are easily indentified. (**b**) Two regions from the same cluster are shown, both having similar local structure; only for better visualization, a sub-graph with 64 nodes is shown in which the local structures around two (similar) vertices are shown. (**c**) Graphlet correlation matrices of an excitatory effective brain network (left panel) and an inhibitory effective brain network (right panel) are shown; only those entries for which correlations (or anti-correlations) are significant (>0.7 and <−0.7) are colored.

Different networks generally have very different graphlet dependencies and hence reveal unknown organizational principles of real-world networks. We study the effective brain networks of 40 healthy subjects. Brain connectivity refers to a pattern of anatomical links (“anatomical connectivity”), of statistical dependencies (“functional connectivity”) or of causal interactions (“effective connectivity”) between distinct units – region of interests (ROI) – within a nervous system^19^. We construct a matrix such that the number of rows in the matrix is equal to the number of vertices in the network and the number of columns equals the dimension of the signature vector (the matrix has 16 columns), see Fig. 2a. The signature vector represents the local structure around the vertex and can be employed to understand organizational rules within a network and to reveal structural differences between networks. We also compute correlation coefficients between all pairs of columns of this matrix and present them in a 16 × 16 symmetric matrix, Fig. 2c.

Degree distribution measures the number of nodes of degree *k*, for each value of *k*. Graphlet degree distribution (GDD) generalizes the notion of the degree distribution: for each orbit *j*, it measures the distribution of the number of nodes “touching” the corresponding graphlet at orbit *j*^32^. It provides a much richer description of topological structure of networks, as the degree distribution is only the GDD of one orbit (the first orbit), which, in the case of directed networks, reflects out-degree, in-degree, and reciprocal-degree distribution. After the nodes are described by GDDs (orbit counts), one can distinguish similar nodes (as defined by any proper distance/similarity metric). Recently, Hočevar and Demšar have analyzed similarity of nodes in an undirected network extracted from the 2013 edition of Wikipedia for Schools^33^. They found that nodes representing countries, cities, and regions have similar orbit counts indicating that for this network it is possible to recognize the nodes corresponding to countries, cities, and regions based on the local network structure represented by GDDs. Graphlets also described local structure of directed networks. Figure 2(b) illustrates that the node/region *cuneus* and node/region *thalmus* are similar (have similar orbit counts). Exploring why the topology around the nodes in the brain network is similar for some regions and not for other regions is beyond the scope of this paper and will be addressed in a future publication. Signature vectors could also be employed to study what/how network properties depend on edge directions by flipping the direction of edges between all connected pairs of nodes with probability one-third (for out-, in-, and reciprocal-edges). This could also be extended to study whether edge directions are relevant for neurodegenerative disorders. Recently, higher-order organization of complex directed networks have been proposed by considering motifs and by introducing generalized framework for clustering networks on the basis of higher-order connectivity patterns^34^. This can easily be generalized to graphlets and/or their orbits: understanding higher-order organization of directed brain networks could be beneficial for understanding mechanisms of neurodegenerative disorders.

### Graphlet correlation

**Figure 3:**
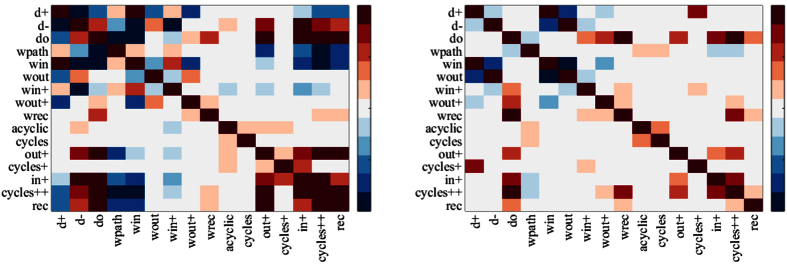
Graphlet correlation matrices are computed for all 40 healthy subjects. Percentages of the healthy subjects that are statistically significant are colored. The correlation is considered significant if the Pearson correlation coefficient is greater than 0.7 and for anti-correlation is considered significant if the coefficient is less than −0.7. The heat-map indicates that there are many pairs of entries of the signature vector that are significantly correlated or anti-correlated for most of the subjects. (**a**) Excitatory effective brain network (**b**) Inhibitory effective brain network.

We then compute the percentage of subjects for which particular coordinates (similarity metrics) of the signature vectors are significantly correlated (>0.7) or anti-correlated (<−0.7), shown in Fig. 3. Since the separation of the positive (excitatory) and negative (inhibitory) connectivity in the effective brain networks, we obtained two matrices which represent the percentage of subjects which contain significant correlation (or anti-correlation) for all graphlet types we take into account.

We found that for excitatory brain effective networks higher values of the number of out-degree is associated (significantly anti-correlated) with lower values of the number of in-degree in 95% subjects and with the number of reciprocal-degree in 64% subjects. Therefore, regions which are involved in activations (G-causes) of other regions (higher values of G-causes manifested with higher values of *d*^+^) at the same time are not activated (G-effects) by other regions (lower values of G-effects shown in both *d*^−^ and *d*°). On the other hand, the number of in-degree and the number of reciprocal-degree are correlated in 62% subjects. Moreover, we found that *W*^(*path*)^ is anti-correlated with *T*^(*out*+)^ (in 74% subjects), *T*^(*cycle*++)^ (in 85% subjects) and with *T*^(*rec*)^ (in 79% subjects). The number of in-wedges *W*^(*in*)^ is anti-correlated with *W*^(*out*+)^ (in 74% subjects), *T*^(*in*+)^ (in 79% subjects), *T*^(*cycle*++)^ (in 85% subjects) and with *T*^(*rec*)^ (in 74% subjects). While the relationships between degrees, wedge-degrees, and wedge-degrees and triangle-degrees are mostly negative (anti-correlated), we found that relationships between different number of triangles (involving reciprocal edges) are positive (correlated): (1) *T*^(*out*+)^ with *T*^(*in*+)^ (69%), *T*^(*cycle*++)^ (100%), *T*^(*rec*)^ (97%); (2) *T*^(*cycle*+)^ with *T*^(*in*+)^ (59%); (3) *T*^(*in*+)^ with *T*^(*cycle*++)^ (100%) and *T*^(*rec*)^ (59%); (4) *T*^(*cycle*++)^ with *T*^(*rec*)^ (100%). However, no significant (positive or negative) relationships are found in which the number of acyclic triangles and the number of cycle triangles are involved.

In general much less significant correlations (positive or negative, anti-correlations) with high percentage overlap are found in inhibitory effective brain networks. We found that *W*^(*in*)^ is anti-correlated with *W*^(*out*)^ in 93% subjects. Moreover, there was 65% correlation overlap between the subjects for graphlets of type *cycles*++ and *in*+. Strong correlation was found between the number of triangles *T*^(*cycles*++)^ and the number of wedges *W*^(*rec*)^ in 68% subjects.

Our study indicates that reciprocal edges are crucial for manifesting correlations between triangle structures. For example, even though triangles are much more correlated between each other (compared to wedges), the two triangle structures that do not have reciprocal edges (*T*^(*acyclic*)^ and *T*^(*cycles*)^) are not correlated to other 3-node graphlet structures. Furthermore, we observed that the correlation between two 3-node graphlets is negative if one of the 3-node graphlets is a wedge, while it is positive only when both 3-node graphlets are triangles. In particular, the local structure *W*^(*path*)^ is anti-correlated with the local structures *T*^(*out*+)^, *T*^(*cycle*++)^, and *T*^(*rec*)^. Also, the local structure *T*^(*out*+)^ is correlated with the local structures *T*^(*in*+)^, *T*^(*cycle*++)^, and *T*^(*rec*)^. Why only two out of six different wedge patterns are significantly anti-correlated with other local patterns (*W*^(*path*)^ and *W*^(*in*)^), and whether this is peculiar for brain networks only, are questions which have yet to be answered. The local structure *W*^(*rec*)^ in effective brain networks is not significantly correlated (or anti-correlated) with any other 3-node graphlet structures. It has been suggested^35^ that in large-scale cortical networks, structural hubs tend to participate in motifs consisting of exactly two reciprocal edges, that is, in the local structure *W*^(*rec*)^. In the same study^35^, a structural network of segregated regions and interregional pathways was obtained from anatomical studies of macaque cortex. Another network, called transfer entropy (TE) network was generated by considering the mean activation of each brain region that was simulated using a nonlinear model of spontaneous neuronal activity. From these activity patterns, maps of interregional interaction were built by measuring transfer entropy in order to capture patterns of directed interaction and information flow. Some of the graph metrics in TE networks are strongly correlated with the metrics in the structural network. Here, we describe graphlet correlation matrices (Fig. 3) in effective brain networks. The questions related to these matrices, their structure, and their relevance for neurodegenerative disorders are beyond the scope of this work and will be discussed in a separate manuscript.

## Conclusion

Vertex signature vector and graphlet correlation matrix are powerful tools for network analysis. We found that the numbers of in, out, and reciprocal neighbors as well as the numbers of local structures (wedges and triangles) are *significantly larger* in excitatory than in inhibitory effective brain networks. By considering only those correlations (or anti correlations) in the correlation matrix that are significant (>0.7 or <−0.7) and are presented in more than 60% of the subjects we found that causal patterns (G-causes and G-effects) are significantly dominant in excitatory compared to inhibitory effective brain networks. However, only one type of local structure is dominant in inhibitory effective brain networks: *i* → *j*, *i* → *k*. In particular, after averaging the aggregated matrix across all subjects and taking into account only ROIs that are outside of the confidence interval (with significance level of 5%), 34.48% from the ROIs have greater number of graphlets members to the isomorphic class *W*^(*out*)^ in the inhibitory network compared to the same nodes in the excitatory network (46.55% from the ROIs have grater number of graphlets in the excitatory network). In the following regions: *middle frontal gyrus, left inferior frontal gyrus (opercular part), left inferior frontal gyrus (triangular part), superior medial frontal gyrus, medial frontal gyrus (orbital part), anterior cingulate and paracingulate gyri, right posterior singulate gyrus, right parahippocamal gyrus, right posterior cingulate gyrus, right parahyppocampal gyrus, left amygdala, calcarine fissure and surrounding cortex, left cuneus, left superior occipital gyrus, left middle occipital gyrus, right postcental gyrus, superior parietal gyrus, inferior parietal gyrus, right supramarginal gyrus, angular gyrus, precuneus, caudate nucleus, right hechl gyrus, left superior temporal gyrus (temporal pole), right middle temporal gyrus, left middle temporal gyrus (temporal pole, hemispheric lobule III, Vermic lobule I/II, Vermic lobule III, Vermic lobule IV/V, Vermic lobule X*, the number of local structure of type *W*^(*out*)^ is greater in the inhibitory network.

This study raises several questions. Network analyses of several neurodegenerative disorders have been well documented^19^,^36^,^37^,^38^. However modeling structural, functional, and effective brain networks as multiplex networks and then using network analytics for understanding different topological features of diseases is an open question yet to be addressed. Moreover, our findings suggest that one could develop exponential random graph models of brain networks across lifespan (longitudinal multimodal MRI Study) - yet another research direction that could further enhance our understanding of how brain connectivity changes in time. How to narrow down the network to describe well defined region or phenomenon in system neuroscience? Is there clinically well-defined area/subsystem where MRI maps well the ROIs to relevant regions/nuclei/tracts? Is the underlying structure directional in local or global signaling patterns? To what extent the excitatory and inhibitory circuits are isolated/measured and characterized somehow? Finally, recent study using ultra-high-field (7T) MRI and spectroscopy has suggested that in humans associative memories are stored in balanced excitatory-inhibitory ensembles^39^. Simulation using a neural network model and replicating experimental steps, these data are shown to be consistent with the balancing of memories via inhibitory synaptic plasticity. How these results are related to our finding that excitatory and inhibitory effective brain networks are not well balanced is the question which we will address in the near future.

## Additional Information

**How to cite this article**: Trpevski, I. *et al*. Graphlet characteristics in directed networks. *Sci. Rep*. **6**, 37057; doi: 10.1038/srep37057 (2016).

**Publisher’s note:** Springer Nature remains neutral with regard to jurisdictional claims in published maps and institutional affiliations.

## Supplementary Material

Supplementary Information
