# Supplementary material for: Graphlet characteristics in directed networks
Source: Sci Rep. 2016 Nov 10;6:37057. doi: 10.1038/srep37057 (PMC5103263; doi:10.1038/srep37057)
Supplement: Supplementary Information [file srep37057-s1.pdf]

Supplementary Information to the paper "Graphlet characteristics in directed networks"

Igor Trpevski[1,+], Tamara Dimitrova[1,+], Tommy Boshkovski[1,+], Nikola Stikov[2,3], and Ljupco Kocarev[1,4,5,*]

[1]Macedonian Academy of Sciences and Arts, Skopje, Republic of Macedonia
[2]Institute for Biomedical Engineering, Ecole Polytechnique, Montreal, QC, Canada
[3]Montreal Heart Institute, Montreal, QC, Canada
[4]Faculty of Computer Science and Engineering, UKIM, Skopje, Republic of Macedonia
[5]BioCircuits Institute, UC San Diego, La Jolla, CA 92093-0402, USA
[*]lkocarev@ucsd.edu
[+]these authors contributed equally to this work

ABSTRACT

Graphlet analysis is part from network theory that does not depend of the choice of the network null model and can provide comprehensive description about local network structure. Here, we offer novel method for graphlet-based analysis of directed networks by computing first the *signature vector* for every vertex in the network and then the *graphlet correlation matrix* of the network. This analysis has been applied on brain effective connectivity networks by considering both direction and sign (inhibitory or excitatory) of underlying directed (effective) connectivity. In particular, the signature vectors for brain regions and the graphlet correlation matrices of the brain effective network are computed for 40 healthy subjects and common dependencies are raveled. We found that the signature vectors (node, wedge, and triangle degrees) are dominant for excitatory effective brain networks. Moreover, by considering only those correlations (or anti correlations) in the correlation matrix that are significant (> 0.7 or < -0.7) and are presented in more than 60% of the subjects, we found that excitatory effective brain networks show stronger causal (measured with Granger causality) patterns (G-causes and G-effects) than inhibitory effective brain networks. Here we provide detailed explanations and provide additional examples to the main paper[1].

1 Graphlet-based similarity

In undirected graphs, there are eight 2- to 4-node graphlets resulting in 15 orbits[2] (of which 11 are non-redundant[2]), that touch a vertex i. Since the number of up to 4-mode graphlets in directed networks is large, here we consider only a subset of these orbits starting (originating) from a vertex. More specifically, let $h_1, ...h_k$, be nodes belonging to graphlets with $2 \leq k \leq 4$ nodes and define $\alpha_1, \alpha_2, \alpha_3, \alpha_4, \alpha_5, \alpha_6 \in \{+, -, \circ\}$. We enumerate all possible ways in which an orbit can touch the node h_1 in these directed graphlets:

$$
\begin{aligned}
O_0(h_1)^{(\alpha_1)} &= S_{h_1}^{\alpha_1} \\
O_1(h_1)^{(\alpha_1,\alpha_2)} &= \left\{h_2, h_3 : h_2 \in S_{h_1}^{\alpha_1}, h_3 \in S_{h_2}^{\alpha_2}\right\} \\
O_2(h_1)^{(\alpha_1,\alpha_2)} &= \left\{h_2, h_3 : h_2 \in S_{h_1}^{\alpha_1}, h_3 \in S_{h_1}^{\alpha_2}\right\} \\
O_3(h_1)^{(\alpha_1,\alpha_2,\alpha_3)} &= \left\{h_2, h_3 : h_2 \in S_{h_1}^{\alpha_1}, h_3 \in S_{h_1}^{\alpha_2} \cap S_{h_2}^{\alpha_3}\right\} \\
O_4(h_1)^{(\alpha_1,\alpha_2,\alpha_3)} &= \left\{h_2, h_3, h_4 : h_{i+1} \in S_{h_i}^{\alpha_i}, i = 1,2,3\right\} \\
O_5(h_1)^{(\alpha_1,\alpha_2,\alpha_3)} &= \left\{h_2, h_3, h_4 : h_2 \in S_{h_1}^{\alpha_1}, h_3 \in S_{h_1}^{\alpha_2}, h_4 \in S_{h_3}^{\alpha_3}\right\} \\
O_6(h_1)^{(\alpha_1,\alpha_2,\alpha_3)} &= \left\{h_2, h_3, h_4 : h_2 \in S_{h_1}^{\alpha_1}, h_3 \in S_{h_2}^{\alpha_2}, h_4 \in S_{h_2}^{\alpha_3}\right\} \\
O_7(h_1)^{(\alpha_1,\alpha_2,\alpha_3)} &= \left\{h_2, h_3, h_4 : h_i \in S_{h_1}^{\alpha_{i-1}}, i = 2,3,4\right\}
\end{aligned}
$$

$$
\begin{aligned}
O_8(h_1)^{(\alpha_1,\alpha_2,\alpha_3,\alpha_4)} &= \left\{h_2,h_3,h_4 : h_{i+1} \in S_{h_i}^{\alpha_i}, i=1,2,3; h_4 \in S_{h_1}^{\alpha_4}\right\} \\
O_9(h_1)^{(\alpha_1,\alpha_2,\alpha_3,\alpha_4)} &= \left\{h_2,h_3,h_4 : h_{i+1} \in S_{h_i}^{\alpha_i}, i=1,2,3; h_4 \in S_{h_2}^{\alpha_4}\right\} \\
O_{10}(h_1)^{(\alpha_1,\alpha_2,\alpha_3,\alpha_4)} &= \left\{h_2,h_3,h_4 : h_{i+1} \in S_{h_i}^{\alpha_i}, i=1,2,3; h_3 \in S_{h_1}^{\alpha_4}\right\} \\
O_{11}(h_1)^{(\alpha_1,\alpha_2,\alpha_3,\alpha_4)} &= \left\{h_2,h_3,h_4 : h_i \in S_{h_1}^{\alpha_{i-1}}, i=2,3,4; h_4 \in S_{h_3}^{\alpha_4}\right\} \\
O_{12}(h_1)^{(\alpha_1,\alpha_2,\alpha_3,\alpha_4,\alpha_5)} &= \left\{h_2,h_3,h_4 : h_{i+1} \in S_{h_i}^{\alpha_i}, i=1,2; h_4 \in S_{h_i}^{\alpha_{i+2}}, i=1,2,3\right\} \\
O_{13}(h_1)^{(\alpha_1,\alpha_2,\alpha_3,\alpha_4,\alpha_5)} &= \left\{h_2,h_3,h_4 : h_2 \in S_{h_1}^{\alpha_1}, h_3 \in S_{h_1}^{\alpha_2}; h_4 \in S_{h_i}^{\alpha_{i+2}}, i=1,2,3\right\} \\
O_{14}(h_1)^{(\alpha_1,\alpha_2,\alpha_3,\alpha_4,\alpha_5,\alpha_6)} &= \left\{h_2,h_3,h_4 : h_2 \in S_{h_1}^{\alpha_1}, h_3 \in S_{h_1}^{\alpha_2} \cap S_{h_2}^{\alpha_3}, h_4 \in S_{h_1}^{\alpha_4} \cap S_{h_2}^{\alpha_5} \cap S_{h_3}^{\alpha_6}\right\}
\end{aligned}
$$

resulting in a total of $1695 = 3 + 2 \times 3^2 + 5 \times 3^3 + 4 \times 3^4 + 2 \times 3^5 + 3^6$ combinations.

Considering only 3-node graphlets and focusing only on orbits which *originate* at node i, we define the following 39 quantities for a node i in the graph:

$$
\begin{aligned}
d_i^\alpha &= |S_i^\alpha| \\
T_i^{(\alpha,\beta,\gamma)} &= \sum_{j \in S_i^\gamma} \left| S_i^\alpha \cap S_j^\beta \right| \\
W_i^{(\alpha,\beta)} &= \sum_{j \neq i} \left| S_i^\alpha \cap S_j^\beta \right| - \sum_\gamma T_i^{(\alpha,\beta,\gamma)}
\end{aligned}
$$

These 39 quantities can be aggregated into 16 quantities by grouping wedges in 6 wedge isomorphic classes and triangles in 7 triangle isomorphic classes, resulting in:

- 3 degrees: d_i^+, d_i^-, and d_i°;
- 6 wedge-degrees:

$$
\begin{aligned}
W_i^{(path)} &= W_i^{(+,-)} + W_i^{(-,+)} \\
W_i^{(in)} &= W_i^{(+,+)} \\
W_i^{(out)} &= W_i^{(-,-)} \\
W_i^{(in+)} &= W_i^{(+,\circ)} + W_i^{(\circ,+)} \\
W_i^{(out+)} &= W_i^{(-,\circ)} + W_i^{(\circ,-)} \\
W_i^{(rec)} &= W_i^{(\circ,\circ)};
\end{aligned}
$$

- 7 triangle-degrees:

$$
\begin{aligned}
T_i^{(acyclic)} &= T_i^{(+,-,+)} + T_i^{(+,+,-)} + T_i^{(+,+,+)} + T_i^{(-,-,-)} + T_i^{(-,-,+)} + T_i^{(-,+,-)} \\
T_i^{(cicles)} &= T_i^{(+,-,-)} + T_i^{(-,+,+)} \\
T_i^{(out+)} &= T_i^{(+,\circ,+)} + T_i^{(-,-,\circ)} + T_i^{(\circ,+,-)} \\
T_i^{(cycles+)} &= T_i^{(+,-,\circ)} + T_i^{(+,\circ,-)} + T_i^{(-,+,\circ)} + T_i^{(-,\circ,+)} + T_i^{(\circ,-,-)} + T_i^{(\circ,+,+)} \\
T_i^{(in+)} &= T_i^{(+,+,\circ)} + T_i^{(-,\circ,-)} + T_i^{(\circ,-,+)} \\
T_i^{(cycle++)} &= T_i^{(+,\circ,\circ)} + T_i^{(-,\circ,\circ)} + T_i^{(\circ,-,\circ)} + T_i^{(\circ,+,\circ)} + T_i^{(\circ,\circ,-)} + T_i^{(\circ,\circ,+)} \\
T_i^{(rec)} &= T_i^{(\circ,\circ,\circ)}.
\end{aligned}
$$

The quantities degrees, wedge-degrees, and triangle-degrees could be normalized in different ways. For example, one can consider normalized signature vector defined as:

$$
\bar{F}_i = \left[\frac{d_i^+}{A_i}, \frac{d_i^-}{A_i}, \frac{d_i^\circ}{A_i}, \frac{W_i^{(path)}}{B_i}, \ldots, \frac{W_i^{(rec)}}{B_i}, \frac{T_i^{(acyclic)}}{C_i}, \ldots, \frac{T_i^{(rec)}}{C_i} \right]^T
$$

where

$$\begin{aligned}
A_i &= d_i^{+} + d_i^{-} + d_i^{\circ} \\
B_i &= W_i^{(path)} + W_i^{(in)} + W_i^{(out)} + W_i^{(in+)} + W_i^{(out+)} + W_i^{(rec)}, \\
C_i &= T_i^{(acyclic)} + T_i^{(cycles)} + T_i^{(out+)} + T_i^{(cycles+)} + T_i^{(in+)} + T_i^{(cycles++)} + T_i^{(rec)}
\end{aligned}$$

By flipping the direction of edges between all connected pairs of nodes with probability one-third (for out-,in-, and reciprocal-edges), which keeps the undirected connectivity and the edge density unchanged, information about the direction of edges will be destroyed and normalized signature vectors should be close to $[\frac{1}{3}, \frac{1}{3}, \frac{1}{3}, \frac{1}{6} \cdots \frac{1}{6}, \frac{1}{7}, \ldots \frac{1}{7}]^T$. This could be further exploited by studying normalized signature vectors for different real-world networks and their distances to random directed network with no direction-generated structure (such graph could be generated by considering arbitrary undirected connected graph and then transforming it to directed graph by assigning to each edge of the graph a direction (out, in, or reciprocal) with probability 1/3).

The number of triangles (but also wedges) could also be normalized as follows. $t_i(\alpha, \beta, \gamma)$ is the normalized number of (α, β, γ) triangles associated with the actor i:

$$t_i(\alpha, \beta, \gamma) = \frac{\sum_{j \in S_i^{\gamma}} |S_i^{\alpha} \cap S_j^{\beta}|}{\sum_{j \neq i} |S_i^{\alpha} \cap S_j^{\beta}|} = \frac{\sum_j x_{\gamma} \sum_h x_{\alpha} x_{\beta}}{\sum_j \sum_h x_{\alpha} x_{\beta}}$$

$\sum_{j \neq i} |S_i^{\alpha} \cap S_j^{\beta}| = \sum_{j \neq i} \sum_h x_{\alpha} x_{\beta}$ is the number of all (α, β) wedges starting at i. Thus, $t_i(\alpha, \beta, \gamma)$ indicates the ratio of (α, β) wedges that are (α, β, γ) triangles. This is a generalization of the concept of clustering coefficient of a graph to clustering coefficient of (α, β, γ) triangle associated to the node i:

$$t_i = \frac{\text{Number of } (\alpha, \beta, \gamma) \text{ triangles}}{\text{Number of } (\alpha, \beta) \text{ wedges}}$$

References

1. I.Trpevski, T.Dimitrova, T.Boshkovski, N.Stikov and L.Kocarev, "Graphlet characteristics in directed networks"
2. O. N. Yaveroğlu, N. Malod-Dognin, D. Davis, Z. Levnajic, V. Janjic, R. Karapandza, A. Stojmirovic & N. Pržulj, "Revealing the Hidden Language of Complex Networks", Scientific Reports 4, Article number: 4547 (2014) doi:10.1038/srep04547
